# West Nile Encephalitis presenting with fever, vertigo, and intention tremor progressing to neurogenic shock and death in Philadelphia area elderly gardener

**DOI:** 10.1016/j.idcr.2026.e02620

**Published:** 2026-05-23

**Authors:** Juan Lopez Tiboni, Carl Kim, Sofia Barrios Fernandez, Ajit Singh, Colin Kulick-Soper, Polly Van Den Berg, Thuonghien Tran

**Affiliations:** a Department of Medicine, Pennsylvania Hospital, United States; b Department of Infectious Diseases, Pennsylvania Hospital, United States; c Department of Pulmonary Critical Care Medicine, Pennsylvania Hospital, United States

**Keywords:** West Nile, Encephalitis, Neurogenic shock, Infectious Diseases, Vector borne diseases

## Abstract

A 77 year old female smoker with history of Polymyalgia Rheumatica and Sarcoidosis presented to hospital with a two day history of fever, nausea, gait ataxia, and new bilateral intention tremor. After admission, she was noted to become rigid with features of parkinsonism and subsequently developed worsening weakness with inability to follow appendicular commands. MRI brain showed midbrain and thalamic infiltration, with CSF findings of markedly elevated WBC, elevated protein, and West Nile IgM. Her condition worsened over the following eleven days to include fulminant flaccid paralysis and neurogenic shock requiring ICU transfer despite empiric treatment with standard of care therapies. Worsening shock refractory to vasopressors, persisting GCS of 3, and rising lactate with acute renal failure prompted family and clinicians to pursue palliative measures. She died with family at bedside 13 days after admission.

## Background

West Nile Virus (WNV) is a relatively uncommon flaviviridae regionally endemic to North America capable of causing significant disease [1], [2]. Infection is driven primarily by bites from the *culex* mosquito but is also possible through contaminated blood products [3], [4], [5]. Despite the vast majority of cases leading to self-limited or mild febrile illness, the most significant associated pathology is neuroinfiltrative disease, occurring in 1 of 150 cases [6], [7], [8], including a 16 fold increase in risk for those age > 65 [9]. Diagnosis of WNV is made through detection of serum West Nile IgM or neuro-invasive disease through CSF IgM [10] and treatment is supportive with no therapies demonstrating benefit when studied [11].

Fatality in patients with WNV encephalitis approaches 10% with advanced age being the most significant risk factor [12]. Initial presentation can include a broad range of febrile symptoms, which can make diagnosis challenging. As the disease progresses from a systemic febrile illness to include neurologic components, diagnosis is often not immediate as other more common diseases take precedence in initial diagnostic pathways. Suspicion for West Nile requires thorough consideration and can be missed entirely. Diagnosis often comes late after other diagnostic avenues and empiric treatments are exhausted or not at all. In cases of severe illness requiring a high level of care, this can often translates to excessive diagnostic procedures, anti-microbials, and other therapies.

This is a case of a 77 year old female who developed neuro-invasive encephalitis in an urban setting. Her disease onset was progressive over the course of 13 days from generalized symptoms to frank neurogenic shock. Despite living in a metropolitan area, identification of key risk factors in her infectious disease history enabled the team to arrive at a diagnosis that prevented unnecessary workup, especially as she progressed to severe illness. Furthermore, this case documents a first in the literature to the authors’ knowledge of WNV induced neurogenic shock. This case serves as an example of severe WNV encephalitis and the tied value of a well taken infectious disease history for other clinicians encountering similar cases.

## Case

A 77 year old female with a history of cerebrovascular accident (2021), borderline Type 2 diabetes mellitus, and polymyalgia rheumatica presented to the emergency department (ED) with chief complaints of fever, dizziness, vomiting, and gait ataxia for three days. On the day of presentation, the patient had severe dizziness and nausea that occurred with any sudden movement of her head causing inability to ambulate, and an localized mild headache. She also endorsed new onset tremulousness in the bilateral limbs, but vestibular symptoms, loss of vision, otalgia, or ear fullness. In the ED, notable labs included a leukocytosis of 11.4 and a urine culture that grew E. coli. An MRI of the head confirmed small acute infarction in the right thalamus with subacute infarct in the right posterior limb of the internal capsule. It was believed that the infarcts were incidental and unrelated to her active presentation. She was admitted with a diagnosis of urosepsis and treated with ceftriaxone.

Over the next two days, despite anti-pyrectics and appropriate antibiotic coverage, she continued to register high fevers, rigors, and, eventually, an altered mental status. Imaging of the abdomen and pelvis ruled out any intra-abdominal abscesses. On closer examination, she had new onset neck stiffness in anterior flexion and rigid stiffness in all extremities. She was subsequently broadened to acyclovir, ceftriaxone, ampicillin, and vancomycin for suspected meningoencephalitis. A lumbar puncture was performed that showed 397 WBCs and 63 RBCs, and an MRI, now done three days after presentation showed multiple new and increasing foci of diffusion in the right thalamus, midbrain, and white matter.

On further questioning of the patient’s social history, she was a very active, highly educated avid gardener with outdoor exposure to mosquitos, a backyard that was inhabited by rodents, and an outdoor cat. She had no documented recent travel outside of the city.

With this information, there was high concern for West Nile Virus (WNV), especially because of its predilection for the basal ganglia and thalami, prompting the team to consider empiric treatment. Though there weren’t any concrete treatment guidelines on West Nile Virus, case series had shown possible benefits with high dose steroids and she underwent a 5 day course of 1000 mg methylprednisolone.

After the course of steroids, she showed improvement, becoming more alert, showing less stiffness, following commands, and speaking with providers and family members. She was also started on carbidopa/levodopa for her cog-wheel rigidity and Parkinsonism examination features.

Five days after her lumbar puncture, her CSF came back positive for West Nile Virus.

After five days of improving clinical status, the patient began to deteriorate again with less responsiveness, inability to move any limbs, no withdrawal to pain, loose muscle tone, and 2 + reflexes in the arms and right patellar and absent in the left patellar and ankle joint. She was restarted on high dose steroids.

She eventually had to be intubated and needed pressor support as she went into neurogenic shock. Though suspicions were low, she was also started on empiric antibiotics for a possible alternate source of sepsis. Her course rapidly deteriorated in the ensuing 24 h in the setting of worsening shock escalating to quadruple pressor therapy, which led to hypo-perfusion mediated lactic acidosis and acute renal failure in the form of anuria, refractory hyperkalemia, and refractory non-gap metabolic acidosis. BMP at this time showed K 5.7 and rising, Cl 115, Bicarb 14, Anion Gap 10, BUN 77, Creatinine 1.07 with a blood gas of pH 1.37, C02 23 (she was set to maximum ventilator settings to compensate her acidosis), p02 91, and a lactate of 6 and rising. Care was withdrawn by family due to evolving organ failure and sustained GCS of 3. The patient was palliatively extubated and expired in hospital with cause of death documented as hypo-perfusion mediated multi-organ failure secondary to neurogenic shock from invasive WNV Encephalitis.

## Discussion

A narrative review of the WNV literature was done to contextualize the disease as it pertains to our case.

The West Nile Virus, first isolated in Uganda in 1937 is one of many viruses in the flaviviridae family. Up until the 1990s, the virus was associated with only mild febrile illness in Northeast Africa and the golden crescent. It was isolated in the Western hemisphere for the first time in 1999, and since then has become an extremely rare but endemic condition throughout Africa, Europe, Southwest Asia, Australia, The Middle East, and the Americas [1]. Interestingly, only the United States and Canada have experienced periodic larger scale outbreaks [2]. Passerine birds are the primary hosts for West Nile, although small mammals and rodents can also act as hosts [13] with human infection typically derived from bites of the *Culex* mosquito [3]. However, the virus can also be transmitted in humans through blood products and organ transplantation [4], [5].

Although the proportion of exposures leading to illness is not well understood, it is thought that among persons that become infected, ∼25% develop fevers and 1 in 150 develop neuroinfiltrative disease [6], [7], [8]. Risk factors for infection were not identified during our literature search. Risk factors for neuro-invasive disease specifically include a 16-fold increase in risk for those age > 65 [9], and an ‘extreme’ increase in risk in those infected through solid organ transplant.

After infection, an incubation period of 2–14 days is typical, although longer periods of up to 21 days have been reported in immunocompromised patients [5], [14]. Symptoms are described as sudden in onset involving headaches, fevers, chills, vomiting, fatigue, malaise, eye pain, and a morbiliform, maculopapular, nonpruritic rash on the torso and limbs sparing the palms and soles [6]. West Nile Meningitis however tends to present as above but with additional meningeal signs and photophobia [15]. Full recovery is the most common outcome in uncomplicated West Nile Fevers and Meningitis [16].

West Nile Encephalitis has a broad range of symptom severity, ranging from self-limiting confusion to severe encephalopathy with extrapyramidal symptoms, parkinsonian features, and coarse tremors in the upper extremities which tend to have postural and kinetic components [16], [17], [18]. Associated paralysis has also been described due to destruction of the anterior horn of the spine, ranging from asymmetric weakness to full flaccid paralysis and quadriplegia [19], [20]. Sensory impairment is notably not described. Other manifestations have been described in the more severe cases, including choroiditis, hepatitis, rhabdomyolysis, locked-in syndrome, Guillan Barre, and autonomic instability [11]. Fatality in patients with WN encephalitis approach 10%, with age > 65 being the most significant risk factor [12].

Diagnosis is made through detection serum West Nile IgM or CSF IgM. Since IgM does not cross the blood brain barrier, detection in the CNS constitutes CNS infection [10]. White cell counts in the blood stream are typically normal, and CSF studies are usually consistent with viral meningitis i.e normal glucose, normal to elevated protein, and lymphocyte predominant pleocytosis [11]. Neuroimaging for invasive disease typically shows no changes, although lesions of the pons, basal ganglia, thalamus, as well as periventricular and leptomeningeal enhancement have been documented [21].

Treatment of West Nile is supportive, with studies trialing IVIG, West Nile-specific monoclonal antibodies, corticosteroids, and interferons all failing to demonstrate benefit [11]. No vaccine currently exists.

This case highlights the potential severity of acute West Nile encephalitis, especially in the setting of exposure to the outdoors in a relatively urbanized area. The patient in this case lived in the Philadelphia area in the US Northeast, but her gardening habits, mosquito exposure, and nearby field rodents were of relevance in the history that prompted the team to investigate for WNV. She was 77, which increased her risk of severe disease. She also presented with an initially nonspecific viral syndrome with associated neurological symptoms that evolved throughout her admission. This case closely follows the illness features that emerged in the literature review.

Additionally, to the authors’ knowledge, this is the first case report in the literature of West Nile Encephalitis advancing to frank neurogenic shock, although we suspect cases like this have occurred but not been submitted for publication given the virus’s known infiltration of the midbrain and reports of autonomic instability documented in review articles [11], [21].

While no curative treatment exists, clinching the West Nile encephalitis diagnosis successfully prevented extensive procedural workup and unnecessary treatments. If anything, this case is a prime example of how a well-taken infectious disease history can be paramount in the diagnosis of an initially cryptic condition causing CNS disturbance.

It is also unclear which role, if any, was played by immune dysregulation in her development of aggressive disease. The literature does not list immunodepleting pharmacotherapy or auto-immune conditions as risk factors for development of more aggressive disease, despite this seeming like a reasonable conclusion to draw based on clinical rationale alone. It is also worth noting the data has not supported any benefit in treatment with immunosuppressive therapy for improving outcomes in moderate or severe disease [11].

In summary, West Nile Encephalitis can progress to parkinsonism, paralysis, and fulminant neurogenic shock. It should remain on the differential when dealing with unspecified CNS disease even in urbanized settings.

## CRediT authorship contribution statement

**Thuonghien Tran:** Writing – review & editing. **Sofia Barrios Fernandez:** Writing – review & editing, Software, Resources. **Ajit Singh:** Writing – review & editing. **Colin Kulick-Soper:** Writing – review & editing. **Polly Van Den Berg:** Writing – review & editing. **Juan Lopez Tiboni:** Writing – review & editing, Writing – original draft. **Carl Kim:** Writing – review & editing, Data curation, Conceptualization.

## Patient consent statement

The patient is deceased and written consent from a legally authorized representative could not be obtained. The details in this case report have been sufficiently anonymized such that the patient cannot be identified. According to University of Pennsylvania IRB policy, a single retrospective case report (of one patient) that is de-identified in this way does not constitute human subjects research and does not require IRB review or patient consent. The report conforms to ethical standards applied in the United States and is consistent with the policy of the University of Pennsylvania.

## Funding sources

None

## Declaration of Competing Interest

None.
